# The politics and imaginary of ‘autonomous vehicles’: a participatory journey

**DOI:** 10.1057/s41599-022-01209-1

**Published:** 2022-08-22

**Authors:** Axelle Van Wynsberghe, Ângela Guimarães Pereira

**Affiliations:** 1 Utrecht University, Utrecht, Netherlands; 2 Joint Research Centre, European Commission, Ispra, Italy

**Keywords:** Social anthropology, Science, technology and society, Social policy, Sociology, Environmental studies

## Abstract

The Connected and Automated Vehicles (CAVs) pilot project set out to explore the alternative mobility imaginaries of participants. These imaginaries challenged the automated vision of the future presented by vehicle and technology companies. This paper takes a post-normal science and digital anthropology approach to the question of automated technology and the role that citizens have in shaping mobility future(s). Through narrative analysis, interviews with stakeholders, and Futures Making Ateliers, this citizen engagement journey deconstructs the technological promises of CAVs, as well as their plausibility and desirability from the point of view of the participants of the participatory journey. Our findings suggest that the technology is solving a different problem than the mobility problem as articulated in policy documents. By investigating the matters of concern of participants, the problem of mobility was redefined in their own terms, and alternative futures were explored. We use the concept of MacGuffin as means to explore the wider relevance of CAVs in mobility futures.

## Introduction

This paper outlines our research on alternative mobility future(s) that were expressed by participants in a series of group futuring activities throughout Europe. Indeed, this multitude of voices speak of futures in the plural—futures that are heterogeneous, that offer critical remarks on technocratic understandings of technology and AI, and that leave spaces for alternatives. Throughout this project, citizens and a variety of other stakeholders whose voices have been side-lined in debates about the role of CAVs in Europe’s mobility future(s) have contributed their own understandings of urban planning, mobility, sustainability, safety, and economic prosperity. This report highlights the often-overlooked gaps and complexities within the discourse that too often espouses a universal vision for the future of urban mobility in Europe and taps into citizens’ imaginaries: their values, expectations, insights and visions.

The European Commission’s Joint Research Centre (JRC) conducted this pilot project, a participatory journey on connected and automated vehicles (CAVs), focused on the social and ethical issues they raise in the context of European policy-making. This pilot was part of the H2020 NewHorrizon project that explores Responsible Research and Innovation (RRI) inspired governance modalities through a number of Social Lab experiments. This particular Social Lab aimed at assessing the potential implications and societal expectations of CAVs and explored various mobility narratives, ethical considerations, expectations and matters of concern toward this new type of mobility. The CAVs pilot, included in-depth interviews with stakeholders on the future(s) of mobility and group settings that we called Futures Making Ateliers which engaged ordinary citizens. It provided the JRC with the opportunity to investigate whether a more persistent and broader RRI approach (Engagement, Ethics, and Governance) could deliver more comprehensive knowledge to sustain policy design. Throughout this project, the imaginaries that the industries are promoting and investing in have been questioned by various stakeholders, which included professionals in the field, but also ordinary citizens. These challenges to the industry’s future mobility proposals have led the JRC to investigate alternative imaginaries of cities and mobility with the participants of our Futures Making Ateliers. We started by looking at the narratives that justified the proposal, anchoring our analysis in the key EU policy document that addresses these innovations: the 2018 Communication of the Commission COM(2018) 237 (European Commission, 2018). In a nutshell, we attempted to deconstruct the social and political purpose of CAVs and explored with citizens alternative mobility futures. Through the deconstruction of existing discourse on the future of mobility, and the critical analysis of the social and technological promises of CAVs, participants could imagine new directions for the future of mobility. In our Futures Making Ateliers, the CAVs functioned as a MacGuffin^1^, a device setting the framework for participants to imagine alternative mobility futures.

Our work is informed by Science and Technology Studies (STS) which critically engages the fields of science and technology and their practices and by post-normal science, which Funtowicz and Ravetz (1993), coined in the 1990s as a problem solving strategy to be used when issues are complex, uncertain, value laden, high stakes and requiring urgent decisions. In PNS an extended peer community needs to be engaged in the framing and in the production of relevant knowledge to address those types of societal issues. Anticipatory governance, which is a way of governing that aims to collect information and data, and analyse these in order to assess possible futures, is informed by foresight, engagement, and the implementation of these activities at the policy-making level (resulting in evidence-based policy). The report ‘Ethics of Connected and Automated Vehicles: Recommendations on road safety, privacy, fairness, explainability and responsibility’ by Bonnefon et al. (2020) underlines the need for the kind of participatory approach to CAVs that we are arguing for in this paper (2020). Digital and design anthropology have also made important contributions that have impacted the ways in which technologies are conceptualised, and have provided methodologies for researching digital culture, tools and infrastructures. Indeed, the ‘sensory and embodied ways of *knowing* and *learning* that characterise everyday experience’ are often overlooked in research that involves emerging technologies (Bonnefon et al. 2020). Concepts such as ‘trust’ and ‘sharing’ used in discourse about CAVs are often taken for granted—instead, they need to be critically examined. Too often, reservations about new technologies are framed as ‘distrust’ or ‘risk-aversion’—relegating their matters of concern to issues of ‘trust’ and ‘acceptance’. We challenge this ‘user acceptance’ framework, which tends to view lack of acceptance as an obstacle to innovation by exploring alternative visions for mobility. This is a term used in the industry to research and analyse obstacles to future users’ acceptance of a technology. Much of the current narratives around CAVs assume that technologies ‘have agency to both engage people in interactional relationships of trust and to change society’ (Pink et al, 2020). Indeed, are the social promises of CAVs not dependent on factors other than the technological promises—what is claimed that CAVs will be able to deliver, such as driverless vehicles? And can they be realised through technological innovation alone? Our empirical work discussed below suggests no for these two questions.

## The social construction of technological artefacts

Connected and automated vehicles (CAVs) are socio-technological artefacts. The term encapsulates the new mobility options that have emerged as a result of innovations in connectivity and Artificial Intelligence (AI) which have opened the door to an ‘Internet of Things’ (IoT). They are vehicles whose functionalities rely on internet connectivity and/or artificial intelligence systems. CAVs are not only shaped by human behaviour but also shape it in return. In other terms, what the automobile and technology sectors term the ‘human factor’ does not disappear when the driver steps away from the wheel. Ethical and political regimes are always-already present in technical artefacts—and that of the CAV sector is that which this paper seeks to address. At its most basic definition, technology can be conceived of as ‘the very ability of humans to treat the world systematically’ (Børsen and Botin, 2014:217). Indeed, techno-anthropology has traced innovations in technology all the way down to the first cognitive leaps that allowed for creativity and language: ‘if you can shape a stone you can shape a sentence’ (MacGregor, 2012:14-15). Furthermore, anthropologists and sociologists have contributed to literature exploring how technological tools ‘do’ things; including how they ‘reproduce the agency of their commissioners, makers and users; (…) evoke emotional reactions within and amongst individuals, and urge people to take certain actions and positions’ (Svasek, 2007:85). Science and Technology Studies has also emphasised the material basis of digital technologies, focusing on the politics of their infrastructures, and standards, rules and norms (Stilgoe, 2017).

The design of digital technologies is a particularly opaque process—not only because the Silicon Valley ideology reinforces the idea of ‘tech geniuses’, but also because structural and political decisions concerning these technologies often occur outside the public eye and are hardly visible once the product is released. Industry anthropologists Erik Vinhuyzen and Melissa Cefkin (Vinhuyzen & Cefkin, 2016) at Nissan state that the main goal for companies developing automated vehicles is that they “ensure ‘socially acceptable’ autonomous driving” (2016). The social dimension of automated vehicles is therefore important to investigate. In *The Social Construction of Technological Systems*, Bijker, Hughes and Pinch (Bijker et al., 2012) frame technological systems as inherently operating in a web of relations dependent on human will and intervention:

“Technological systems, even after prolonged growth and consolidation, do not become autonomous; they acquire momentum. They have a mass of technical and organisational components; they possess direction, or goals; and they display a rate of growth suggesting velocity. A high level of momentum often causes observers to assume that a technological system has become autonomous. (…) The large mass of a technological system arises especially from the organisations and people committed by various interests in the system.” (2012: 70)

Rather than humans being ‘liberated from the driving task’, the act of driving becomes mediated by a technological system, itself upheld by various types of human labour. Indeed, as Cefkin states, autonomy itself ‘is an abstraction, it does not exist in an absolute sense (…) The interplay with people will remain, we just have to see exactly where and in what ways’ (The human side of autonomous cars, 2017). Several authors have meaningfully re-conceptualised the human-car relationship. In Dant’s work, for example, the driver-car has been analysed as ‘a form of social being that produces a range of social actions’ such as polluting and killing (2004). In that of Brown and Laurier, the task of driving has also been examined as a ‘social activity’ (2017). A lot of what automated vehicles strive to address, such as road traffic, can also be conceived of as social in nature, as it is ‘a broad mixture of people [finding] their way to various destinations using a variety of transportation options, ultimately, and nearly unavoidably, by means of interacting with other road users to establish a self-organised order of traffic’ (Vinhuyzen and Cefkin, 2016:523). The driver is oftentimes subjected to a host of external circumstances to anticipate how others move, such as weather or time of day. Even rules of the road are subject to a driver’s judgement and/or social awareness, and tacit knowledge is often used to decide where and when certain rules must be followed, and how.

The AV will not only have to take social cues—it will also have to give them. Drivers often signal to other vehicles or pedestrians in order to communicate movement of the vehicle. Human drivers are constantly interacting with one another through many small encounters and making important decisions as a result of these—such as whether to yield at an intersection, gauge apparent erratic driving from young or elderly drivers, and anticipate situations involving pedestrians. Autonomous cars must be versed in these encounters if they will ever succeed. Nevertheless, it is clear that vehicles have also already altered urban infrastructure and social behaviour in turn (Pink et al., 2019). It is because of this that the implementation of new technological innovations must be examined closely, especially in the case of CAVs where their deployment would radically alter not only how road infrastructure is built and cities are constructed, but also how people move, work and play—or even how we conceive of space, time, and community.

## Methodology

Throughout the pilot project, we conducted narrative analysis, in depth interviews with stakeholders, and group conversations with citizens across Europe that we called Futures Making Ateliers (FMA). As Chilvers & Kearnes (2015) note, public engagement is not just about exploring opinions and interests, but about openly discussing matters of concern. This engagement exercise, therefore, focused on collecting participants’ insights, expectations, values, and visions rather than their ‘opinions’.

The narrative analysis was conducted using core texts—books, journal articles, and news articles—contributing to the discourse on CAVs and determining (1) the social and ethical issues raised, (2) the promises of the technology, and (3) the technical issues raised (Van Wynsberghe & Guimarães Pereira, 2021a). From this work emerged the main technological and social promises of CAVs that we used throughout our citizen engagement activities. This work was vital to map the current discourse on CAVs and identify the stakeholders involved, some of which we approached for our interviews. This was a necessary step to validate our work on the identification of narratives and to extend it with their knowledge on the issue. It would also enable us to determine and validate the social and technological promises currently in the field and their plausibility from the point of view of relevant stakeholders, including policymakers.

Semi-structured interviews were conducted with nine interviewees within the technology and automobile sectors. Our interviewees worked in a variety of fields such as ITS, electronics engineering, road infrastructure, safety, and research and innovation, amongst others. Our interviewees were also based in many countries across the EU, such as the Netherlands, Belgium, Portugal, the United Kingdom, and Italy. The transcriptions of the interviews were analysed according to this coding framework: (1) Technological Promises and Challenges, (2) User Acceptance and Experience, (3) Governance, (4) Connectivity and Autonomy, (5) Energy and Sustainability. In order for our interviewees to remain anonymous, our interviewees will be referred to as: Interviewee A, B, C, D, E, F, G, H, I [Appendix A].

The Futures Making Ateliers held by the JRC were an opportunity for participants to explore and co-create future scenarios regarding EU mobility. We term them in this manner because of the critical engagement with possible futures (in this case, CAVs that have achieved Level 5 automation), and the engagement with speculative futuring techniques. We also use the plural term ‘futures’ to question and de-centre narratives promoting one particular future and to explore a variety of scenarios with participants. We held eight of such futuring settings across Europe with 148 participants in total [Appendix B]. The JRC ensured that these were held in local languages, and in various countries in Europe: Italy, Portugal, and Belgium. The participants were from Greece, Italy, Portugal, Austria, Poland, Belgium, the Netherlands, France, and the United Kingdom. Several of our futuring activities were conducted during EU Regions week, which further included a wide demographic of stakeholders. The Futures Making Ateliers’ length was 3 h, with the exception of those held during EU Regions week - see Van Wynsberghe & Guimarães Pereira, 2021b). The interpersonal, political, and civic capacities of participants may differ; the JRC, therefore, aimed to offer enough contextual information, whilst also allowing space for in-depth debates within and between groups. In citizen engagement activities, there is no claim towards representativeness—the aim is rather to gain a deeper understanding on the issues and critically engage with the framework and research questions.

**Fig. 1:**
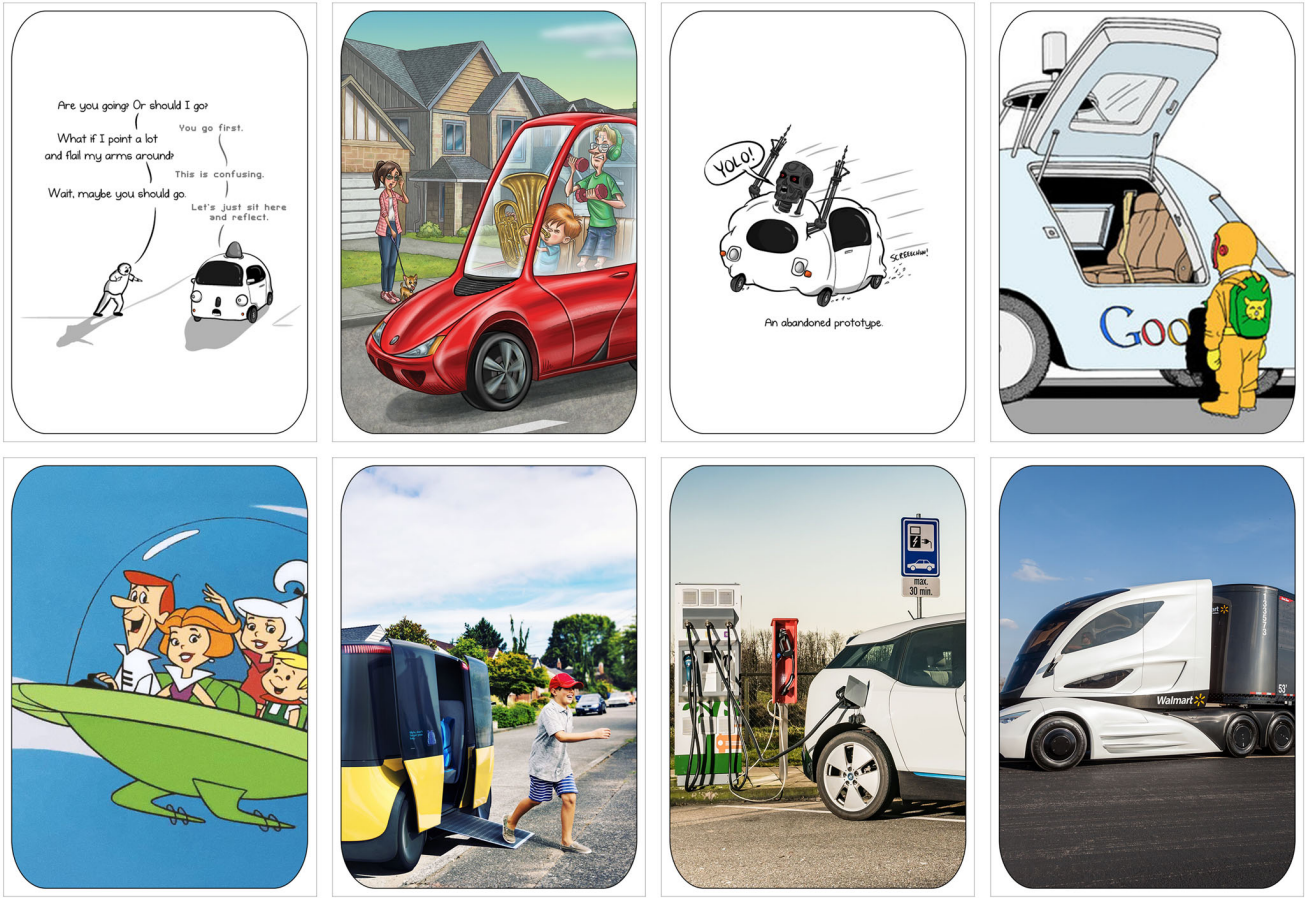
CAV cards visual. Visual of ‘CAV Cards’ developed by the Joint Research Centre’s (JRC) Connected and Automated Vehicles (CAVs) pilot project with imagery from several public websites.

The Futures Making Ateliers included several activities that were heavily influenced by material deliberation (Davies et al. 2012), which is a participatory methodological approach through which material and affective knowledge that has insofar been excluded from expert and policy debates on policy issues can be valued and included. Indeed, we specify ‘atelier’ because of the use of objects through which material deliberation could take place, and the settings in which we conducted the research, which mostly included Makerspaces designed for these types of activities. Throughout this process, iterative and inclusive participatory multi-actor dialogues were established between researchers, policy makers, industry and civil society organisations, NGOs, and citizens. The use of objects—through demonstrations, card games and ^®^Lego^2^—during the futuring settings made them accessible to a variety of demographics such as children and young adults and allowed for alternative forms of knowledge to be included in discussions about CAVs. We created our own ‘CAV Cards’—see Fig. 1 – that contained various representations of CAVs—these included popular references such as *The Jetsons*, or *Back to the Future*, as well as industry advertising, or artistic depictions of social and ethical concerns. The cards that we used, in contrast to the ‘AD Futures’ cards proposed by Pink et al., did not use any form of text (2020). The transcriptions of the conversations held during the participatory journey were also analysed according to this coding framework: (1) Technological Promises and Challenges, (2) User Experience, (3) Social and Ethical Issues, (4) Governance, (5) Connectivity and Autonomy, (6) Energy and Sustainability. The transcripts were additionally analysed in order to determine the types of vehicles mentioned, types of energy sources mentioned, and the types of ownership mentioned, as well as the occurrence of utterances of each category.

**Fig. 2:**
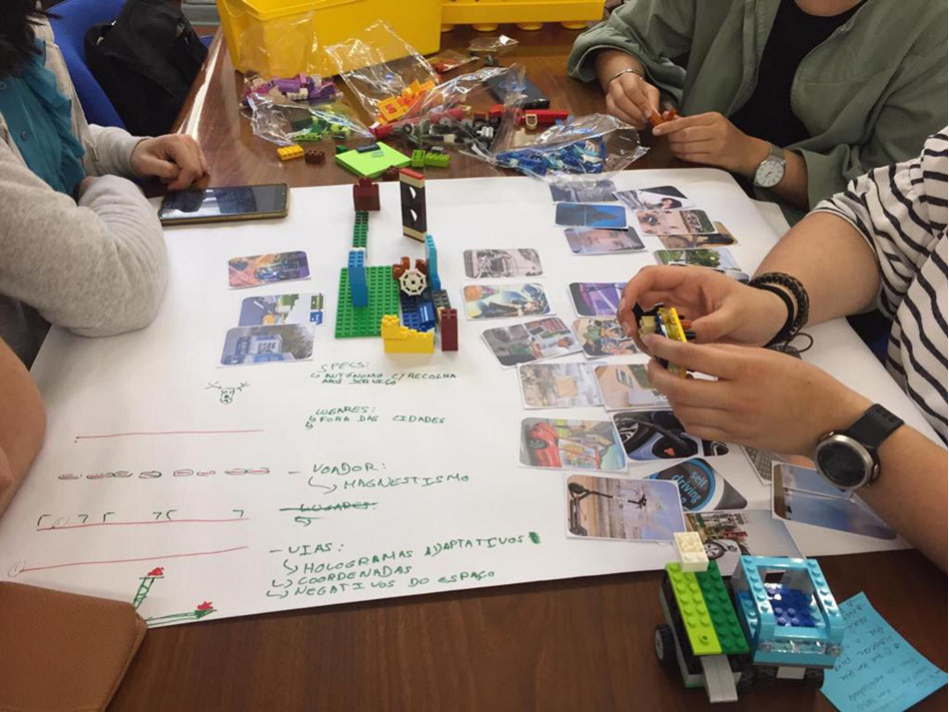
Photo of material deliberation. Photo of material co-creation and deliberation through ®Lego and ‘CAV Cards’ at a Futures Making Atelier in Lisbon.

Firstly, in ‘Narratives of Mobility’, participants were asked to respond to statements pulled from the European Commission’s Communication ‘On the Road to Automated Mobility: An EU Strategy for Mobility of the Future’ (2018). Once presented with statements about CAVs and the future of mobility, they were to explain whether these claims appeared a) plausible and b) desirable to them. ‘CAV Cards’ depicting the various aspects and issues of CAVs were distributed to the participants in order to engage discussion. Secondly, in ‘Vehicles of the Future’, participants were asked to create vehicles with ® Lego building blocks and answer the question: ‘What mobility problems do you solve with that vehicle?’ [Appendix C]. The ^®^Lego was used to prototype an answer to the questions asked. These ‘prototype’ vehicles were used to elicit a discussion on the participants’ matters of concern as well as allow for them to critically engage with the socio-technological artefact of CAVs. Once they showcased their vehicles, the moderator asked each participant in addition to what problem their vehicle solves, also who the vehicle is for. Thirdly, in ‘Imagining a Neighbourhood’, participants were tasked to illustrate their future(s) of mobility in groups—see Fig. 2. Participants had to consider the implication of their choices on issues of safety, sustainability, data and privacy, user agency, implementation as well as the urban environment and available infrastructure.

The citizen engagement methodologies that we used to engage our participants allowed for them to question the narratives, promises and framings put forth by a variety of actors, including industry actors and the European Commission. This created space for participants to shape the conversation on their terms and provide thoughtful and reflexive interventions. It allowed participants to appropriate the discussion on the future(s) of mobility and tailor it to their social, cultural and geographical context. Due to this, we were able to collect a wide array of mobility futures rather than maintain superficial consensus on one version of the future of mobility as espoused by current policy makers pushing for futures spearheaded by innovations in automation.

## Troubling mobility future(s): the social and technological promises of automated vehicles

Technology and automobile companies, as well as actors within the policy sector, frequently state that CAVs will be revolutionising society. One journal article states that the emergence of driverless technology is considered an ‘unprecedented revolution in how people move’, whilst another positions automated vehicles as ‘enhancing common wellbeing and prosperity’ (Daziano et al. 2017; Cohen and Cavoli, 2019). Questions such as: ‘What are you doing to seize the CAV opportunity to better meet your city’s mobility objectives and improve quality of life?’ and ‘Who’s driving your future?’ can be observed explicitly or implicitly being posed to citizens, policymakers and city officials in the literature (Batten, 2019).

The promises about CAVs that have been identified throughout our narrative analysis fall into two main categories: (1) technological promises, concerning the performance of CAV technology, and (2) social promises, referring to perceived social benefits as the outcome of its implementation. The social promises are often framed as a direct result from the technological promises of CAVs, thereby framing the technology itself as the ‘solution’ to central issues in the European Union. Our interviewees, which featured key players in the industry, shed light on the capacity of the automobile and technology sectors to deliver on technological promises concerning CAVs. Their responses complicated and/or questioned the promises that are being used to justify the implementation of this technology; such as that CAVs will be safer, more sustainable, and accessible than current mobility options.

“In many of those visions a miracle happens, and all the cars are automated and connected from one day to the other. In reality, we have millions and millions of cars in Europe and the exchange of the cars will be very slow and this technology can only, let’s say, drain into the fleet with new cars. You cannot actually retrofit existing cars with automation, so that will take a very, very long time actually to come to the fleet.” [Interviewee H]

“The incentive alignment is wrong, because it’s incentivising individual car ownership, so that needs to be a different, and car manufacturers need to be incentivised through governance and through various financial incentives, to do things differently.” [Interviewee I]

“Cities are not necessarily opening their arms to connected and automated vehicles and then to that I would say—why should they? I mean, what’s the benefit for them? If they don’t see a value, then they’re certainly not going to facilitate or enable it. (…) When it comes to connected and automated vehicles, there are just so many uncertainties about when they’ll be there, what the different levels of automation^3^ are, when it will really have an impact on the way which people behave, and on the overall mobility of a city.” [Interviewee G]

“You can have an opportunity from let’s say the environmental point of view, and I’m not so convinced that we will have one because I’m not sure that this kind of technology will reduce the mileage that will be travelled by people. Probably it’s exactly the opposite (…) For instance, if you start operating trucks on a platooning let’s say mode of operation, this is something that will impact and is expected to have maybe 16%, 15% or reduction in the consumption of fuel on each truck. And then this is very important for the truck industry, but it is even important for the environment of course and sustainability. But, maybe even the traffic is increasing.” [Interviewee D]

“If we are talking about private cars, then it’s most likely that sophisticated technologies will be introduced to high-priced executive cars in the beginning. So, probably it will not be cheap, it’s for those people who can afford such a high-priced car.” [Interviewee H]

Furthermore, the interviews highlighted the lack of voices from city-officials and citizens. One of the key assumptions being made was that users will eventually ‘accept’ the technology once it is released:

“My personal impression is that as soon as any such technology actually is at their hands, consumers very quickly embrace it. They may have concerns beforehand, but as soon as they actually let’s say, can touch it and they can use it, then they very soon understand the benefits they can have from that and then they very quickly embrace it.” [Interviewee H]

Despite the assumptions that we found and the gaps in the plausibility and desirability of CAVs, policy-makers—including those at the European Commission—are often reproducing the same narratives as that of the automobile and technology industries. The European Commission’s communication is entitled ‘On the Road to Automated Mobility: An EU Strategy for Mobility of the Future’ (European Commission, 2018). The title already insinuates a future of automated mobility, and one that focuses on road mobility. The communication reproduces many of the promises about what this technology will accomplish: ‘it is anticipated that driverless mobility will decrease transport costs, free driver’s time, and foster car sharing, thereby improving air quality and urban planning.’ It frames the future benefits of automated mobility according to perceived benefits that users will reap: ‘[it’s a] new level of cooperation between road users which could potentially bring enormous benefits for them and for the mobility system as a whole, including making transport safer, more accessible and sustainable.’ The central promise of CAVs is to reduce if not eliminate road fatalities caused by ‘human error’. ‘Vision Zero’—zero road fatalities—is posited by the European Commission as the ultimate goal. The communication also highlights importance of reducing pollution and addressing congestion. The deployment of driverless mobility is ‘expected to contribute significantly to achieving these key societal objectives.’ (2018: 2).

Participants within the Futures Making Ateliers questioned whether it was plausible or even desirable to eliminate the human factor in driving.“

“We asked if the human factor is only negative. If we are going to eliminate it, it does not say if it is totally or partially, but it says to eliminate (…)” [FMA1, 30/05/2019]

They also commented on the plausibility of liberating citizens from the ‘driving task’, and of reducing road fatalities through automated vehicles.

“The free time of the driver, in the sense that the transport time will be the same, all the driver has to do is read the newspaper on board, it is not that it takes less to do the transport, so the free time must be read in a restrictive. He runs the risk that (…) he is reading and crashes, as happened in the [Tesla] accident mentioned above.” [FMA7, 14/10/2019]

Referring to the phrase ‘It is imperative to introduce autonomous driving technology’ in the EU communication, a participant illustrated the results of the discussion they had.

“It is not autonomous cars that will save fewer people just because they are autonomous cars, it is the awareness of drivers that saves lives or not. (…) We consider that the fact that it is a self-driving car is not as perfect of an idea. Because there are several variables in the car, for example, my colleague was describing it in terms of deciding who dies and who lives. Someone will always die and as long as you are a driver with some driving experience, you can save people.” [FMA1, 30/05/2019]

Participants also debated the level of agency that users would be able to have in a fully automated vehicle.

“It must be autonomous, but it must not be automatic. (…) It must increase our capacities, it must not be a substitute. In other words, it must empower, it must not be a disabling factor in making decisions, that is, it must not decide for [the user].” [Participants, FMA1, 30/05/2019]

The affective dimension was brought into current debates over liability and its legal framework in the case of an accident caused by an automated vehicle.

“How do you deal with guilt? If there was an accident, who is to blame? The person who programmed it? The person who sent you forward? The government that made the legislation?” [FMA1, 30/05/2019]

Some participants showcased desirability for CAVs, but others struggled to see a clear need that the CAVs would fulfil.

“I am completely out of having automatic driving, because I don’t see this need.” [FMA7, 14/10/2019]

Many participants spoke of “the great issues of digital privacy, digital rights” that CAVs necessarily pose, and the risks that citizens and the transportation system itself run when this information is not secured. The risks of hacking and terrorism concerning the technology used in CAVs were also mentioned.

“As for connected, I am absolutely against it, because the internet is one of the most insecure places we know. I am from the old school that says that the only safe disk is the one in the closet, because when it is on the network it is no longer safe (…) I see the connection of the machines as a crazy danger (…) Not even dead, I don’t get on a connected car. Lots of public industries are blackmailed that they have to pay for computers, let alone what can happen when you screw up millions of cars in Milan bumping into each other (…) this can never be done in my opinion.” [FMA6, 14/06/2019]

It was interesting to see some participants reframe the conversation about the technology—often framed according to ‘trust’ and ‘acceptance’ in terms of governance. In this case, a participant re-centres the conversation on the technicians and engineers rather than on the vehicles.

“I think that people trust technology a lot but maybe they do not trust technicians.” [FMA4, 17/06/2019]

CAVs may strive to ‘eliminate the human factor’, but human attention and intervention is needed for almost all levels of existing and potential automation, with only the possibility to ‘free drivers’ once Level 5—‘full automation’—is reached^4^. But automated vehicles, even at level 5, will have to function within particular predetermined parameters in order to function optimally.

The technological and social promises of CAVs may have identified and propose to fulfil certain societal needs—such as to reduce if not eliminate fatal road accidents, to reduce emissions, heighten traffic efficiency, increase public space, free time, and safety—rely on many other global and situated factors in order to be realised. Like any other big idea, CAVs may help making sense of innovation and in this case also reflect on how this particular innovation can bring any betterment for people, cities, mobility, energy, and sustainability. As we have discussed earlier though, the promises associated to CAVs cannot be actualised through the deployment of the technology alone. The technological promises emerging from the technology and automobile industries remain uncertain due to challenges that have presented themselves over the course of the development and testing stages of CAVs. The social promises, being dependent on the first category, can therefore be considered doubly uncertain, due to the additional complexities that surround the implementation of connected and automated vehicles.

Not only are the social promises of CAVs dependent on the actualisation of their technological promises, but the plausibility and desirability of these promises must continue to be questioned by researchers and policy-makers. These promises are additionally predicated on many assumptions, such as that: CAVs will need little to no human input; they are driven by social needs like road and driver safety; and perceived benefits of AV far outweigh perceived risks. Their development and implementation also threaten to create a plethora of new needs. Some of them include the need to retrain workers in the driving sector, the need for new legislation around data, privacy and liability, and the need for stronger protections against cyber attacks on our transport infrastructure. Attempts to eliminate car accidents through CAVs may also cause new categories of accidents due to the system’s own vulnerabilities.

## Alternative mobility futures for europe

“You have offered the future of mobility as connected and automated vehicles in the communication of 2018—but you have to open it up because it doesn’t resonate with people, their needs, and what they know about how they move.” [WC-FMA, 9/10/2019]

The plausibility and desirability of the technological and social promises of CAVs have been questioned throughout this paper—but why are these promises so enticing? The discourse on CAVs seems to open up a space to reimagine the future of mobility; it represents exciting, almost magical, possibilities. No matter their potential deployment and implementation, CAVs can be seen as an opportunity to (re)imagine the way we move and the infrastructures that are currently in place. In our ateliers, the topic of CAVs opened up a wide array of issues for participants concerning their mobility futures. Through our speculative futuring activities, participants created their own neighbourhoods for the future.

**Fig. 3:**
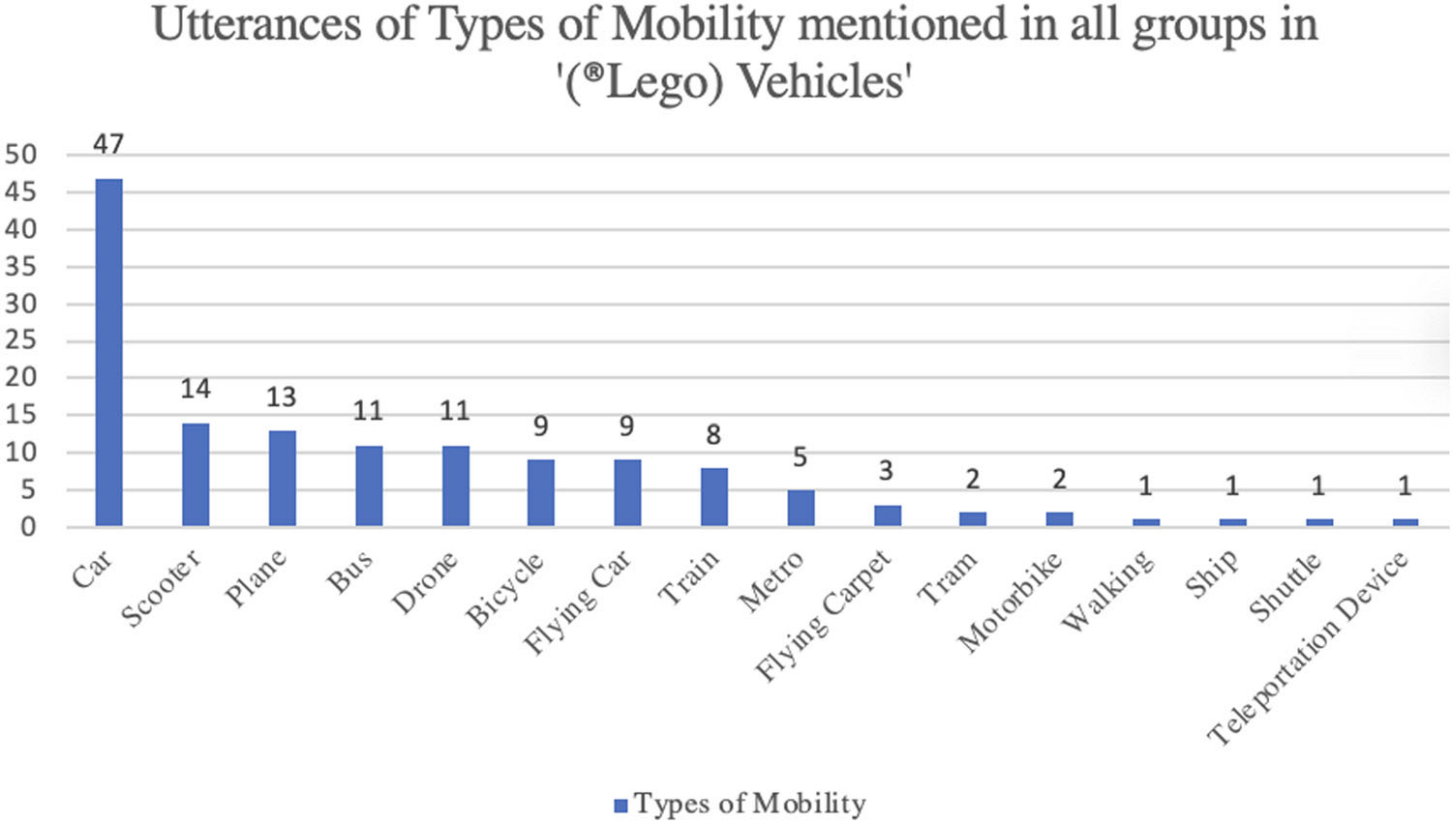
Graph of utterances of types of mobility. Graph of utterances of types of mobility mentioned in all groups when referring to their ®Lego vehicles.

When participants made their own (^®^Lego) vehicles of the future, they emphasised ones that were ‘multimodal’, ‘versatile’, ‘flexible’, ‘adapted’, and ‘easy’, showcasing a desire for vehicles that have multiple functions—for example, for public and private transport, and/or for long and short journeys. They also used words such as ‘modular’ and ‘transform’ to illustrate the variety of functionalities that their vehicle contained. They used a variety of verbs that indicated new types of air, underground and underwater transport, such as ‘hover’, ‘fly’, and ‘levitate’. Most participants spoke of their vehicles being shared in some way—whether they were publicly or privately owned. They mostly created private vehicles, however, which could have the option of becoming shared. This trend changed in the group activity, when participants were asked to imagine their neighbourhoods in the future—these visions included more public forms of transportation. The types of mobility that engendered the most conversation and debate were cars, bicycles and walking – see Fig. 3. Forms of public transportation such as the metro, tram and bus were present in many imaginaries, and many alternative options such as flying taxis and carpets, as well as personal drones, were mentioned. The main problems that participants wanted to solve with their vehicles were: the inefficiencies of public transport, congestion, accessibility, ›pollution, climate change, lack of space and safety.

The matters of concern of participants were illustrated through their imaginaries on the future of mobility. The visions of groups differed, but in co-creating neighbourhoods of the future with other participants, several key factors in their decision-making were identified:

- The safety and security of passengers as well as pedestrians and other citizens not using CAVs
- The ability of their imagined vehicles to meet sustainability goals and tackle climate breakdown, as well as how such vehicles and infrastructures would fare in future climate disasters
- How accessible their vehicles were to certain demographics, such as children and the elderly
- What resources were needed to build and power their imagined vehicles, and the impact of these on communities both in Europe and abroad
- How their decisions affected the digital and privacy rights of citizens in general
- The direct economic benefit of the decisions they were making for workers and users
- The maintenance of the user’s sense of agency within the vehicles

Several trends can be observed in the future visions created by the participants. Firstly, in building their alternative neighbourhoods, many groups focused on how to use space in a different way to change the mobility needs of a city, and they shifted their focus from individual and private mobility options to more public and shared options. New ideas for urban development, such as building vertical forests and denser city spaces with closer services, were brainstormed. Secondly, the impact of climate change on future cities was a recurring theme; they mention concerns about future weather conditions and the loss of flora and fauna. Thirdly, many groups focused on limiting or eliminating the need for journeys requiring (connected and/or automated) vehicles, and even sought to eliminate existing vehicles from circulation. Fourthly, automation was most often spoken about in correlation to other forms of public or shared transport, such as buses, taxis and metros. Adjectives such as ‘swarm’ were used to describe a novel type of connected (and possibly also automated) form of transportation that allows for personalised mobility options through a shared or public service that does not have a fixed route. Lastly, safety concerns were focused on the dangers of hacking, mixed driving and lack of attention from users in automated vehicles, rather than simply on curbing accidents.

It is important to note that these explorations of the future of mobility did not always include or prioritise movement or the introduction of new, more efficient, vehicles.

“I think the issue for us was not so much autonomous or not but cars or not. We want to get rid of the car.” [FMA2, 12/06/2019]

“Although we can use the cars as before, it is no longer necessary.” [FMA1, 30/05/2019]

“Rather than pushing for the different types of automated vehicle, we looked at trying to eliminate the need for movement.” [FMA3, 14/06/2019]

In their visions for future neighbourhoods, many groups focused on clearing space within their urban environments:

“We would never have car parks or garages inside our city, but outside in a space delegated for that.” [FMA1, 30/05/2019]

“We even hypothesised that there were no buildings, but we didn’t reach any conclusion.” [FMA1, 30/05/2019]

Increasing mobility was also not the priority of all participants. Several questioned the need for heightened mobility.

“Will mobility be as necessary as it is today? In my opinion not, because I see it with my children, who at least 20% of their time, unlike us, (…) work through their mobile office, which is the tablet, the mobile phone, etc. I imagine that there will be an ever-greater evolution towards this type of activity which therefore requires less mobility and less need to be in a vehicle whether automatic or not.” [FMA6, 14/10/2019]

Indeed, as this participant mentions, it is perhaps more likely to imagine a future in which our conception of work and technologies shift our lifestyles, than one in which vehicles become fully automated and entrusted with human lives. This participant also notes that although mobility is often divided into the separate categories of travelling for ‘leisure’ or ‘work’, but these are not ‘two things that travel in parallel,’ but are rather ‘complementary’ [FMA6, 14/10/2019].

Although participants explored many different sources of energy, alternative sources of energy that are ethically resourced and sustainable were emphasised. The types of fuels mentioned were Gasoline (5), Diesel (5), and Ethanol (2). Participants also explicitly mentioned human power when referring to active modes of transport, as well as new innovations in magnet technologies and plant energy. Electricity was most mentioned, most likely due to the premise of electric vehicles being related to one of the core promises of CAVs—more sustainable transport. These data from our Futures Making Ateliers correlates with current trends of decreasing coal (by almost 8%), oil (by almost 5%) and gas (by almost 2%) demand. In contrast, ‘Renewables were the only source that posted a growth in demand, driven by larger installed capacity and priority dispatch’ (IEA, 2020).

Due to the narrative analysis work that was conducted, and the way in which a critical and reflexive engagement with these was fostered throughout our citizen engagement activities, participants felt able to contribute to the main debates concerning CAVs. In the ‘(Lego) Vehicle’ and ‘Future Neighbourhood’ activities, participants actively questioned each other on perceived assumptions and biases, exchanged their own knowledge, and evaluated trade-offs. The material deliberation that was fostered in the ateliers helped to illustrate the future(s) of citizens and their main matters of concern. The salient outcome of this journey has been that the technology functioned as a **MacGuffin**, in other words, it was the prompt that enabled participants to imagine alternative mobility futures akin with their imaginaries. Such engagement activities should play a central role in policy-making involving technological innovations, as these results showcase how participants’ experiences can allow for a variety of outcomes and solutions to be considered without falling prey to technological ‘solutionism’. As Bonnefon et al. (2020) state, policymakers ‘have the responsibility to foster active public engagement and facilitate the involvement of all stakeholders for responsible innovation of CAV technology’. CAVs are catering to particular societal needs, but they are not, nor can they be, the only solution in addressing them.

## Conclusion

Through narrative analysis, semi-structured interviews with stakeholders, and Futures Making Ateliers with citizens, this pilot project assessed the plausibility and desirability of the main technological and social promises related to CAVs and the future of mobility. Through citizen engagement methodologies, including material deliberation and speculative futuring, citizens’ imaginaries of the future(s) of mobility were collected. These futures challenged assumptions made by other stakeholders and explored alternatives featuring active modes of transport, low tech solutions and behavioural and/or infrastructural changes that could impact how we move in the first place.

Our results also showcased that there remain important technological, social and ethical challenges to be addressed in the development of CAVs. Our research critically demonstrated how crucial it is to involve citizens at early stages of policy development, not only because of outcomes which often challenge predominant narratives about innovation and technology, but also because our findings suggest that the technology is aimed at solving a different problem than mobility problems, as articulated in policy documents. Our (albeit limited) empirical work suggests that this policy document was not properly informed by citizens’ concerns about their current mobility problems and their expectations. Indeed, the social and technological promises that were identified in the CAV field were not considered plausible or desirable by most participants. The Futures Making Ateliers also challenged certain industry notions of citizen ‘distrust’ of technology and the perceived success of disruptive technologies. By investigating the various issues that impact or could impact citizen lives in relation to CAVs, we move beyond the technology (technological solutions) as the sole imaginary to address mobility issues and into a broader understanding of the role of governance in addressing mobility, and the potential role of citizens in shaping our futures. There are many different types of sectors of society that would be impacted by policy on CAVs and their potential implementation. The mobility futures promoted by policy-makers and industry do not only concern the transport sector; they will play an intrinsic role in how citizens imagine themselves in relation to others and their cities, countries, even the world. Most of all, we can expect that there will not be a ready-made single mobility future to set to policy—rather, they will necessarily be emergent and ‘always in the making’ as European citizens, city officials, mechanics and technologists trial new mobility options across Europe (Ingold, 2013). It is, therefore, necessary to keep bringing new voices into these debates—voices from cities, from rural areas, other areas of policy and from citizens. Indeed, in this study, CAVs served as a MacGuffin which revealed the matters of concern of participants which redefined the problem of mobility in their own terms and explored alternative futures.

At the time we carried out this research, the COVID-19 pandemic had not yet begun. It has, in certain ways, highlighted the interdependencies between transport and other areas of life. It has also questioned what the mobility needs of citizens actually are, and opened up the possibility for alternative solutions beyond CAVs (e.g., changes in urban infrastructure). Indeed, there is no other time in which the human factor has been placed more evidently at the core of city planning and technological innovation. The health crisis has now drastically impacted transport behaviour in Europe. Upstream participatory approaches to policy-making are critical at this time in order to address uncertainty about what mobility will look like in the coming decades, and what kind of alternative future(s) we can imagine. In general, such interventions are crucial in order to examine and question the ‘solutionist’ narratives that surround emerging technologies.

Although this social lab was not anchored in the EU communication development process, our analysis illustrates how this type of work is necessary in order to develop fit for purpose policies. In October 2021, the European Commission launched a Competence Centre on Participatory and Deliberative Democracy (CC-DEMOS)^5^ which is a significant step to institutionalise citizen engagement practices and make the social labs type of engagement more routine when it comes to designing EU policy. One of the outputs from this social lab is a guiding tool that helps with implementing *responsible* knowledge management which informs policymaking [Van Wynsberghe et al. 2022 forthcoming]. Finally, this work suggests that engaging the *extended peer community* in policymaking is the way to work with *extended* framings and seek for a more *extended* body of knowledge to properly inform and design fit for purpose policies.

## Supplementary information

Appendices

## Data Availability

The data generated by the interviews and Ateliers cannot be made available as per informed consent signed by the authors and participants. The participants were informed that the transcripts and audio materials would be destroyed upon completion of the research.

## Footnotes

1 In fiction, a MacGuffin (sometimes McGuffin) is an object, device, or event that is necessary to the plot and the motivation of the characters, but is also ultimately insignificant, unimportant, or irrelevant in itself, existing mainly to spur action within the story. See Wikipedia.

2 There is plenty of literature on how to use Legos in participatory events. For more complex use of Lego in participatory activities, see for example Lego Serious Play.

3 Levels of automation of vehicles can broadly be defined as: Level 0: No Driving Automation; Level 1: Driver Assistance; Level 2: Partial Driving Automation; Level 3: Conditional Driving Automation; Level 4: High Driving Automation; Level 5: Full Driving Automation (Taxonomy and Definitions for Terms Related to Driving Automation Systems for On-Road Motor Vehicles - SAE International, 2021). In this participatory journey we were focusing on the Level 5.

4 In fact, also Level 4 allows the possibility to “free drivers”.

5 https://knowledge4policy.ec.europa.eu/participatory-democracy/about_en.
