# Supplementary material for: The politics and imaginary of ‘autonomous vehicles’: a participatory journey
Source: Humanit Soc Sci Commun. 2022 Aug 22;9(1):284. doi: 10.1057/s41599-022-01209-1 (PMC9395839; doi:10.1057/s41599-022-01209-1)
Supplement: Supplementary file 1 — Appendices [file 41599_2022_1209_MOESM1_ESM.docx]

**Appendix**

**Appendix A: Interviewees**

| Interviewee | Sector | Background & Expertise |
| --- | --- | --- |
| A | Business | Cooperation for research and innovation, Robert Bosch GmbH |
| B | Non-Profit | Political scientist, the *Fédération Internationale de l’Automobile* (FIA) |
| C | Business | Automotive electronics engineer, designing electronic systems for cars made in Europe and US Allianz group |
| D | Business | Road infrastructure and the implementation of CAVs project, deployment of European programs (project manager of the European ITS platform), SINA |
| E | Business | Navigation and pre-GPS systems in the US and Europe, consultation for companies such as Jaguar Land Rover, for Nissan, Toyota, and Volvo |
| F | Supra-National  Organisation | ITS, traffic management systems, connected and automated driving, professor and national expert for DG RTD at the European Commission |
| G | Non-Governmental Organisation | Thematic working group on traffic conditions, POLIS |
| H | Automobile Association | Transport policy, traffic issues focusing on the digitisation of transport and automation at *Allgemeiner Deutscher Automobil-Club* (ADAC) |
| I | Non-Governmental Organisation | Responsible research and innovation, public dialogues concerning technology governance |

**Appendix B: Futures Making Ateliers**

| **Futures Making Ateliers and Location** | **Date** | **Location and Language** | **Number of Participants** | **Methodology** |
| --- | --- | --- | --- | --- |
| **FMA1: Makers, FabLab Benfica,**  **Lisbon (mix. ages)** | 30 May 2019 | Lisbon, Portugal –  in Portuguese | 8 | Futures Making Ateliers |
| **FMA2: JRC Trainees,**  **makerspace JRC (<30 y.o.)** | 12 June 2019 | Ispra, Italy – in  English | 6 | Futures Making Ateliers |
| **FMA3: Children & Families,**  **makerspace JRC (mix. ages)** | 14 June 2019 | Ispra, Italy – in  Italian | 13 | Futures Making Ateliers |
| **FMA4: JRC Staff, makerspace**  **JRC (mix. ages)** | 17 June 2019 | Ispra, Italy – in  English | 9 | Futures Making Ateliers |
| **FMA5: European School**  **students (15-16 y.o.),**  **makerspace JRC** | 18 June 2019 | Ispra, Italy – in  English | 13 | Futures Making Ateliers |
| **FMA6: Makers, WeMake**  **FabLab, Milan (mix. ages)** | 14 June 2019 | Milan, Italy – in  Italian | 9 | Futures Making Ateliers |
| **WC-FMA: Future Urban Mobility:**  **mobilising different**  **knowledges in the debate -**  **EU Regions Week (mix. ages)** | 9 October 2019 | Brussels, Belgium  – in English | 80 [divided into groups of 6] | Futures Making Ateliers |
| **FMA7: Older citizens, MSLD**  **makerspace Milan (>60 y.o.)** | 14 October  2019 | Milan, Italy – in  Italian | 10 | Futures Making Ateliers |

**Appendix C: Results of ‘(®Lego) Vehicle’ Activity**

futures making atelier 1

| Vehicle | Type of Vehicle | Type of Mobility | Functionality | Energy | User(s) | Problem(s) it addresses |
| --- | --- | --- | --- | --- | --- | --- |
| A | Flying Carpet | Shared | Multimodal, in the form of a tanker and used to clear air pollution | CO2 and Magnets | Individual and adaptable for living | Energy consumption, pollution, climate change, parking |
| B | Train | Public | Multimodal | Unavailable | Commuters and flyers, with amenities to eat and work | Flying within Europe |
| C | Bus | Individual, shared | Privately-owned vehicle with sharing option | Unavailable | Commuters for short journeys | Lack of connection to the outside, restriction of vehicles to roads |
| D | Bus | Public | Driverless and automated personalised transport | Unavailable | Commuters | Limited routes of public transport, traffic and carpooling inefficiencies |
| E | Car | Private | Small private vehicle, flexible for families | Renewable energy (hydrogen, electricity) | Families | Practical and traffic problems in cities |
| F | Car/Aeroplane | Shared | Small aerial vehicle | Unavailable | Commuters | Global warming, pollution, speed |

futures making atelier 2

| Vehicle | Type of Vehicle | Type of Mobility | Functionality | Energy | User(s) | Problem(s) it addresses |
| --- | --- | --- | --- | --- | --- | --- |
| A | Car | Private, Public Options | Car-sharing option, with individual and shared parts of the modular vehicle | Unavailable | Families | Commuting |
| B | Truck | Public, Shared | Collector for personalised mobility | Electric | Workers | Storage, space |
| C | Flying Car | Public or Private, Shared | Driverless flying car with shield generator that protects from collisions | Electric | Taxi-users | Accidents |

futures making atelier 3

| Vehicle | Type of Vehicle | Type of Mobility | Functionality | Energy | User(s) | Problem(s) it addresses |
| --- | --- | --- | --- | --- | --- | --- |
| A | Pod | Public, Shared | Automated, driverless pod | Electric | Commuters that usually bike or walk in a world with teleworking | Commuting |
| B | Car | Private | Self-driving square modular flat car that expands with added users | Electric | Individuals and families | Travel, rigidity |
| C | Bicycle | Private | Flexible foldable bicycle to commute and access public transport | Electric | Individuals | Population, storage, flexibility |

futures making atelier 4

| Vehicle | Type of Vehicle | Type of Mobility | Functionality | Energy | User(s) | Problem(s) it addresses |
| --- | --- | --- | --- | --- | --- | --- |
| A | Car | Private | Small aesthetic racing car resembling a wheelchair | Unavailable | Individual | Traffic, congestion, parking, attractiveness |
| B | Bus | Public, Shared | Fast large ground vehicle for storage and speedy commutes | Electric or Fuel | Group of commuters, families | Alternative to fast trains, storage |
| C | Flying Car | Public or Private | Small flying vehicle | Unavailable | Commuters | Congestion, lack of space, population |
| D | Flying Car | Private or Public, Shared | Modular hover car, with possibility for individual or shared use | Unavailable | Individual | Flexibility |
| E | Car | Private | Flying car that can go underwater | Renewables | Group of commuters | Pollution, storage |

futures making atelier 5

| Vehicle | Type of Vehicle | Type of Mobility | Functionality | Energy | User(s) | Problem(s) it addresses |
| --- | --- | --- | --- | --- | --- | --- |
| A | Elevator | Public | Swarm of vehicles offering personalised mobility | Electric or Solar | Commuters | Individual ownership, rigid and inefficient routes |
| B | Flying Car | Public or Private | Aerial vehicle | Fuel | Commuters | Lack of space, traffic, accidents |
| C | Bus | Public | Accessible bus to collect commuters | Plant | Commuters | Individual ownership and commuting, climate change |

futures making atelier 6

| Vehicle | Type of Vehicle | Type of Mobility | Functionality | Energy | User(s) | Problem(s) it addresses |
| --- | --- | --- | --- | --- | --- | --- |
| A | Car | Private, Individual | Automated vehicle | Unavailable | Group of commuters | None |
| B | Car | Private or Public, Shared | Multiple controls and types of engines depending on the driver | Renewables | Individual(s) | Agency and safety within an automated vehicle |
| C | Car | Shared | A technologically evolved sharing platform | Electric or Solar | Group of commuters, individual | Agency and safety within an automated vehicle |
| D | Car | Public, Shared | Starter vehicle, or novel form of public transport | Unavailable | Commuters | Inefficiencies of public transport, traffic |
| E | Train | Public, Shared | Magnetic levitating vehicle | Magnets | Commuters | Lack of funding for existing public transport |
| F | Drone | Private or Public | Flying drones | Electric | Commuter(s) | Congestion, traffic, connection between cities and suburbs |
| G | Car | Private or Public | Automated vehicle as a people’s aid | Unavailable | Disabled people, commuters | Accessibility, speed, agency |
| H | Teleportation Device | Private or Public | Teleportation | Unavailable | Commuters | Energy, pollution, climate change, |

futures making atelier 7

| Vehicle | Type of Vehicle | Type of Mobility | Functionality | Energy | User(s) | Problem(s) it addresses |
| --- | --- | --- | --- | --- | --- | --- |
| A | Car | Private or Public | Vehicle in mixed driving environment | Unavailable | Commuters | Safety, speed |
